# Supplementary material for: HIVprotI: an integrated web based platform for prediction and design of HIV proteins inhibitors
Source: J Cheminform. 2018 Mar 9;10:12. doi: 10.1186/s13321-018-0266-y (PMC5845081; doi:10.1186/s13321-018-0266-y)
Supplement: Supplementary file 2 — Additional file 2. Source code of HIVProtI web server. [file 13321_2018_266_MOESM2_ESM.zip › HIVprotI_Source-code/hivprotI/weka-3-6-6/doc/weka/classifiers/pmml/consumer/NeuralNetwork.html]

NeuralNetwork


---


|  |  |  |
| --- | --- | --- |
| **Overview** \| **Package** \| **Class** \| **Tree** \| **Deprecated** \| **Index** \| **Help** \| **Weka's home** | |  |
| **PREV CLASS**   **NEXT CLASS** | **FRAMES**    **NO FRAMES**     **All Classes** |  |
| SUMMARY: NESTED \| FIELD \| CONSTR \| METHOD | DETAIL: FIELD \| CONSTR \| METHOD |  |


---


## weka.classifiers.pmml.consumer Class NeuralNetwork

```
java.lang.Object
  weka.classifiers.Classifier
      weka.classifiers.pmml.consumer.PMMLClassifier
          weka.classifiers.pmml.consumer.NeuralNetwork
```

**All Implemented Interfaces:**: java.io.Serializable, java.lang.Cloneable, CapabilitiesHandler, OptionHandler, PMMLModel, RevisionHandler

---

``` public class NeuralNetwork extends PMMLClassifier ```

Class implementing import of PMML Neural Network model. Can be used as a Weka
classifier for prediction (buildClassifier() raises an Exception).

**Version:**
:   $Revision 1.0 $

**Author:**
:   Mark Hall (mhall{[at]}pentaho{[dot]}com)

**See Also:**: Serialized Form

---

**Constructor Summary**

`NeuralNetwork(org.w3c.dom.Element model,
Instances dataDictionary,
MiningSchema miningSchema)`


| **Method Summary** | |
| --- | --- |
| `double[]` | `distributionForInstance(Instance inst)`             Classifies the given test instance. |
| `java.lang.String` | `getRevision()`             Returns the revision string. |
| `java.lang.String` | `toString()` |

**Methods inherited from class weka.classifiers.pmml.consumer.PMMLClassifier**

`buildClassifier, done, getCreatorApplication, getDataDictionary, getFieldsMappingString, getLog, getMiningSchema, getPMMLVersion, mapToMiningSchema, setCreatorApplication, setLog, setPMMLVersion`

**Methods inherited from class weka.classifiers.Classifier**

`classifyInstance, debugTipText, forName, getCapabilities, getDebug, getOptions, listOptions, makeCopies, makeCopy, setDebug, setOptions`

**Methods inherited from class java.lang.Object**

`equals, getClass, hashCode, notify, notifyAll, wait, wait, wait`

**Constructor Detail**

### NeuralNetwork

```
public NeuralNetwork(org.w3c.dom.Element model,
                     Instances dataDictionary,
                     MiningSchema miningSchema)
              throws java.lang.Exception
```

**Throws:**: `java.lang.Exception`


**Method Detail**

### getRevision

```
public java.lang.String getRevision()
```

:   **Description copied from class: `Classifier`**
:   Returns the revision string.

    :   **Specified by:**: `getRevision` in interface `RevisionHandler` **Overrides:**: `getRevision` in class `Classifier`
    :   **Returns:**: the revision

---


### distributionForInstance

```
public double[] distributionForInstance(Instance inst)
                                 throws java.lang.Exception
```

:   Classifies the given test instance. The instance has to belong to a
    dataset when it's being classified.

    :   **Overrides:**: `distributionForInstance` in class `Classifier`
    :   **Parameters:**: `inst` - the instance to be classified **Returns:**: the predicted most likely class for the instance or Instance.missingValue() if no prediction is made **Throws:**: `java.lang.Exception` - if an error occurred during the prediction

---


### toString

```
public java.lang.String toString()
```

:   **Overrides:**: `toString` in class `java.lang.Object`


---


|  |  |  |
| --- | --- | --- |
| **Overview** \| **Package** \| **Class** \| **Tree** \| **Deprecated** \| **Index** \| **Help** \| **Weka's home** | |  |
| **PREV CLASS**   **NEXT CLASS** | **FRAMES**    **NO FRAMES**     **All Classes** |  |
| SUMMARY: NESTED \| FIELD \| CONSTR \| METHOD | DETAIL: FIELD \| CONSTR \| METHOD |  |


---
